# Clueless is a conserved ribonucleoprotein that binds the ribosome at the mitochondrial outer membrane

**DOI:** 10.1242/bio.015313

**Published:** 2016-02-01

**Authors:** Aditya Sen, Rachel T. Cox

**Affiliations:** Department of Biochemistry and Molecular Biology, Uniformed Services University, Bethesda, MD 20814, USA

**Keywords:** Mitochondria, Clueless, *Drosophila*, Ribosome, Ribonucleoprotein

## Abstract

Mitochondrial function is tied to the nucleus, in that hundreds of proteins encoded by nuclear genes must be imported into mitochondria. While post-translational import is fairly well understood, emerging evidence supports that mitochondrial site-specific import, or co-translational import, also occurs. However, the mechanism and the extent to which it is used are not fully understood. We have previously shown Clueless (Clu), a conserved multi-domain protein, associates with mitochondrial outer membrane proteins, including Translocase of outer membrane 20, and genetically and physically interacts with the PINK1–Parkin pathway. The human ortholog of Clu, Cluh, was shown to bind nuclear-encoded mitochondrially destined mRNAs. Here we identify the conserved tetratricopeptide domain of Clu as predominantly responsible for binding mRNA. In addition, we show Clu interacts with the ribosome at the mitochondrial outer membrane. Taken together, these data support a model whereby Clu binds to and mitochondrially targets mRNAs to facilitate mRNA localization to the outer mitochondrial membrane, potentially for site-specific or co-translational import. This role may link the presence of efficient mitochondrial protein import to mitochondrial quality control through the PINK1–Parkin pathway.

## Abstract (precis)

**Summary:** Clueless is a ribonucleoprotein that is crucial for mitochondrial function. Here we show Clueless can bind the ribosome at the mitochondrial outer membrane, suggesting it may play a role in mitochondrial co-translational import.

## INTRODUCTION

Mitochondria have a complex life cycle in the cytoplasm. They cannot be made *de novo* and must replicate, undergo transport along the cytoskeleton, and undergo fission and fusion to change shape (Mishra and Chan, 2014). The cell relies on mitochondria not just for ATP production, but also for the production of many important metabolites and molecules, such as those derived from beta-oxidation and heme biosynthesis. Defects in any of these processes can lead to disease and tissue dysfunction, with certain cell types such as neurons and myocytes being particularly sensitive to reductions in mitochondrial capacities due to their heavy reliance on ATP concentrations and calcium buffering. Like cellular components, mitochondrial DNA, protein complexes and membranes can become damaged through oxidation and other means. The cell has evolved mechanisms that can degrade damaged components either selectively, or by targeting specific mitochondria for destruction via a specialized autophagic mechanism known as mitophagy.

Mitochondria have their own DNA, mtDNA, which encodes for proteins used in the complexes of the electron transport chain. However, due to the evolved symbiotic relationship between mitochondria and the nucleus, there are hundreds of proteins encoded in the nucleus that need to be imported into the mitochondrion in order for the organelle to carry out all of its biochemical reactions. Nuclear-encoded mitochondrial destined mRNAs are translated in the cytoplasm where the resulting proteins then bind chaperones which escort them to the mitochondrial translocase apparatus at the mitochondrial outer membrane (Gabriel and Pfanner, 2007). However, there is increasing evidence that site-specific mRNA translation, even co-translational import, occurs at the mitochondrial outer membrane (Eliyahu et al., 2010; Gerber et al., 2004; Kellems et al., 1975; Verner, 1993).

The protein Clueless (Clu) is a large, multi-domain protein that is highly conserved and directly required for mitochondrial function (Cox and Spradling, 2009; Sen et al., 2013, 2015). Lack of Clu causes mitochondrial mislocalization in *Drosophila* cells as well as a severe drop in ATP production and mitochondrial oxidative damage (Cox and Spradling, 2009; Sen et al., 2013). These effects appear to be direct because Clu can associate with several mitochondrial outer membrane proteins, including porin and Translocase of outer membrane 20 (TOM20) (Sen et al., 2015). In addition, Clu genetically and biochemically interacts with the PINK1–Parkin complex, suggesting it not only has a role in supporting mitochondrial function, but can also play a role in mitochondrial quality control (Cox and Spradling, 2009; Sen et al., 2015). Yeast Clu1p has been shown to bind mRNA, and the human Clu ortholog, Cluh, has been shown to preferentially bind nuclear-encoded mitochondrially destined mRNAs (Gao et al., 2014; Mitchell et al., 2013). Here, we show *Drosophila* Clu can also bind mRNA. Using a structure–function approach, we show only the N-terminal-most domain of Clu is dispensable for function *in vivo* and in cell culture, and that the tetratricopeptide repeat (TPR) domain is responsible for binding mRNA. We also show Clu binds five ribosomal proteins and two Eukaryotic initiation factor (eIF) 3 components and that these proteins are highly expressed in a manner similar to Clu in larval stem cells called neuroblasts, cells that undergo rapid divisions during larval growth. Previously, we showed Clu is found in both mitochondrial and cytoplasmic fractions (Sen et al., 2015). Here we show Clu is able to form a complex with the large Ribosomal protein 7a only in the mitochondrial fraction. Taken together, these data support a model whereby Clu is involved in binding and directing nuclear-encoded mitochondrially destined mRNAs to the mitochondrial outer membrane and potentially positioning them for co-translational import. Perturbations in the role of Clu in this function may be used as a sensor to link protein import to the PINK1–Parkin mitophagy pathway (Sen et al., 2015).

## RESULTS

### Multiple domains of Clueless are required for function

**Fig. 1.**
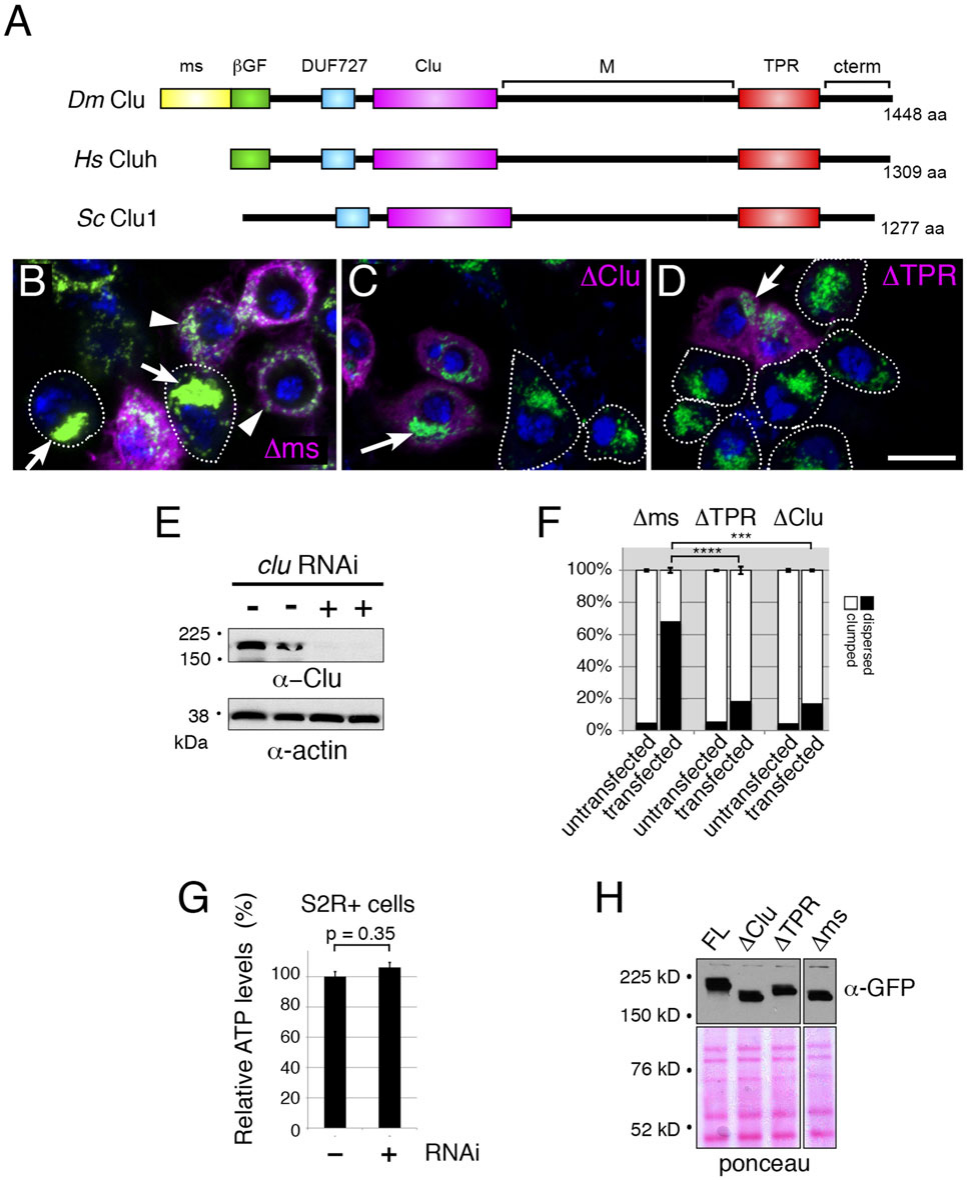
**The Clu and TPR domains are required for Clu function *in vitro*.** (A) Schematic comparing *Drosophila* (*Dm*), Human (*Hs*) and yeast (*Sc*) Clu proteins. Yeast Clu1p lacks the β grasp fold domain (βGF, green) and both human and yeast lack the melanogaster specific domain (ms, yellow). TPR, tetratricopeptide repeats; M, middle domain. (B-D) Exposing S2R+ cells to *clu* RNAi causes the normally dispersed mitochondria to clump (arrows, B). Transfecting *clu* RNAi-exposed cells with GFP-tagged *clu* lacking the ms domain (Δms) disperses mitochondria and rescues this phenotype (B, magenta, arrowheads) relative to non-transfected cells (B, arrows). Transfecting with GFP-tagged *clu* lacking either the Clu domain (ΔClu, C) or TPR domain (ΔTPR, D) does not rescue the mitochondrial clumping phenotype (magenta, arrows). (E) Exposing cells to *clu* RNAi effectively decreases the amount of Clu as judged by western blot. (F) Quantification of the percentage clumped and unclumped mitochondria observed in transfected and untransfected cells in B-D. (G) S2R+ cells with reduced Clu do not have significantly reduced ATP levels relative to control cells. (H) Western blot showing levels of each construct transfected into S2R+ cells. Ponceau serves as a loading control. Lanes are from the same blot. Green=anti-CVA, magenta=anti-GFP, blue=DAPI for B-D. Scale bar=10 µm. Error bars indicate standard error of mean. *P*-values were calculated in Excel using a two-tailed Student's *t*-test. ***P<0.00005, ****P<0.000007.

Clu has close orthologs in many species, including human and yeast, and contains multiple putative domains based on sequence alignment within species, as well as literature searches (Fig. 1A) (Cox and Spradling, 2009; Sen et al., 2015). However, we do not know how each putative domain contributes to the role of Clu in the cell. To better understand which domains are important for its cellular function, we deleted various domains then reintroduced the deletion constructs into *clu*-RNAi knockdown S2R+ cells or flies lacking *clu*. S2R+ cells exposed to *clu*-RNAi have greatly reduced Clu protein as judged by Western blot, and form tightly clumped mitochondria in the cells (Fig. 1E,B, arrows) (Sen et al., 2015). Previously, we showed the clumping phenotype is rescued by transfecting with either full-length Clu (FL-Clu) or the human ortholog of Clu (Cluh; Sen et al., 2015). Transfecting *clu*-RNAi exposed cells with a GFP-tagged *clu* construct lacking the ms domain (Δms) causes mitochondria to disperse to the same degree as we have previously shown with FL-Clu, (Fig. 1B, arrowheads; Fig. 1F). However, expressing GFP-tagged Clu proteins lacking the Clu (ΔClu) or TPR (ΔTPR) domains did not rescue the clumping phenotype very well, indicating these domains are necessary for proper mitochondrial localization (Fig. 1C,D, arrows; Fig. 1F). While depleting Clu in S2R+ cells causes a very dramatic redistribution of mitochondria, it does not reduce ATP levels in the cells (Fig. 1G). This could be because cultured cell lines can have altered metabolism due to transformation and/or exposure to glucose from the culture medium (e.g. Rodriguez-Enriquez et al., 2001, see Discussion for further elaboration). All of the constructs expressed protein at similar levels and at the correct sizes (Fig. 1H).

**Fig. 2.**
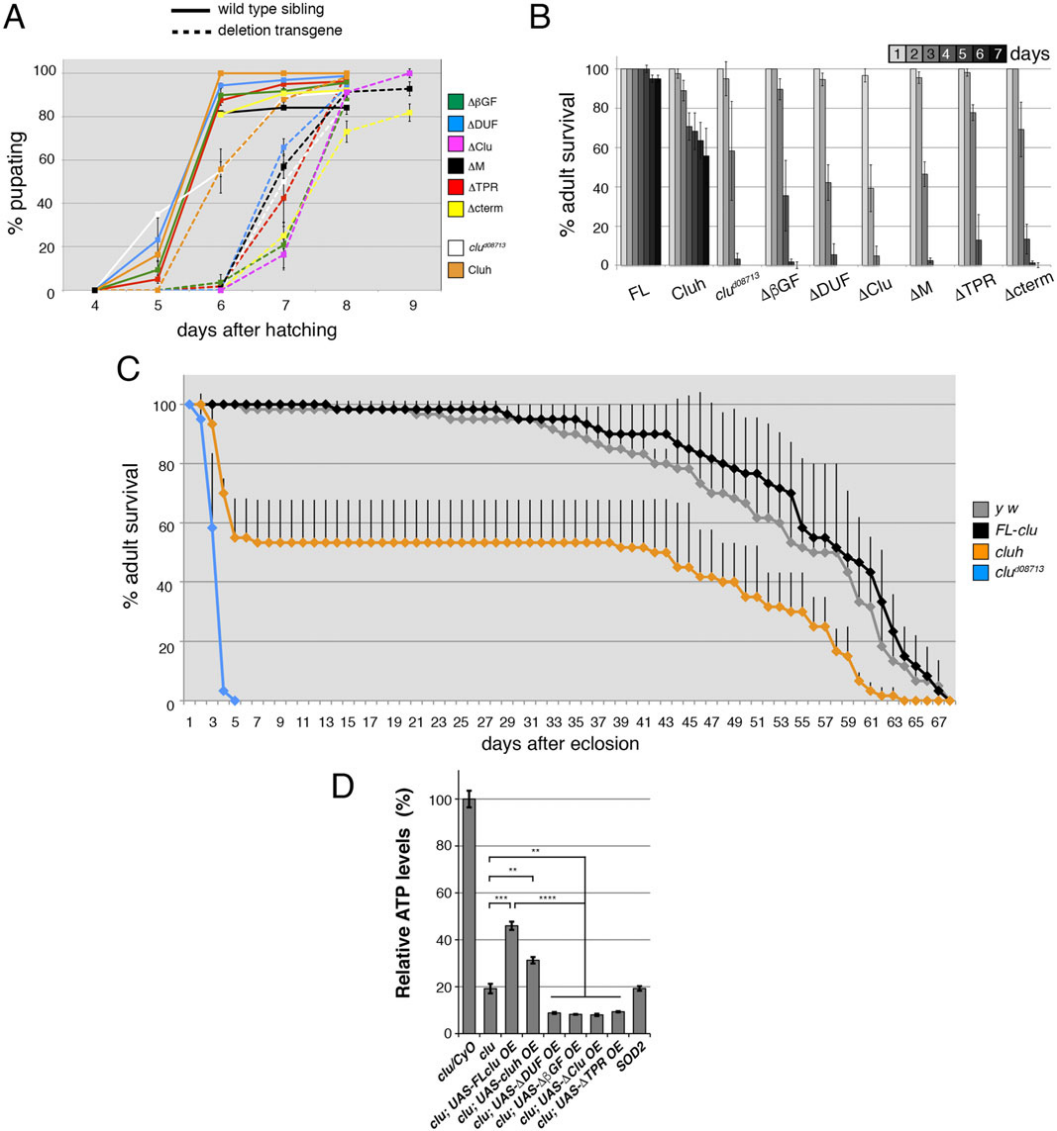
**The Clu and TPR domains are required for Clu function *in vivo*.** (A) Expressing UAS-*clu* domain deletion constructs in a *clu* null background fails to rescue the delay in pupation. Dashed lines represent larvae expressing each deletion construct under the control of *daGAL4* (*clu^d08713^*; *daGAL4*/UAS-transgene). The color-matched solid lines represent the wild-type siblings as a control (*clu^d08713^*/CyO ActGFP; *daGAL4*/UAS-transgene). *clu^d08713^* (orange) and *clu^d08713^*; *daGAL4*/UAS-*cluh* (white) are negative and positive controls for pupation rate, respectively. (B) UAS-driven Clu constructs missing each putative domain fail to rescue adult survival when ectopically expressed in a *clu* mutant background using *daGAL4*. (C) Survival of *clu^d08713^*; *daGAL4*/UAS-*cluh* adults stabilizes after a week, and the remaining flies live a normal lifespan (orange line). *y w* (grey) and *clu^d08713^*; *daGAL4*/UAS-*FL-clu* (black) are positive controls and *clu^d08713^* (white) is a negative control. (D) Adult ATP levels normalized to *clu* heterozygous flies. Adult flies over-expressing full-length Clu (FL) using *daGAL4* have significantly increased ATP levels compared to *clu* null adult flies. Adult flies over-expressing ΔDUF, ΔBGF, ΔClu and ΔTPR using *daGAL4* do not show any increase in ATP levels. Error bars indicate standard error of mean. *P*-values were calculated in Excel using a two-tailed Student's *t*-test. **P<0.008, ***P<0.0006, ****P<0.00004.

**Fig. 3.**
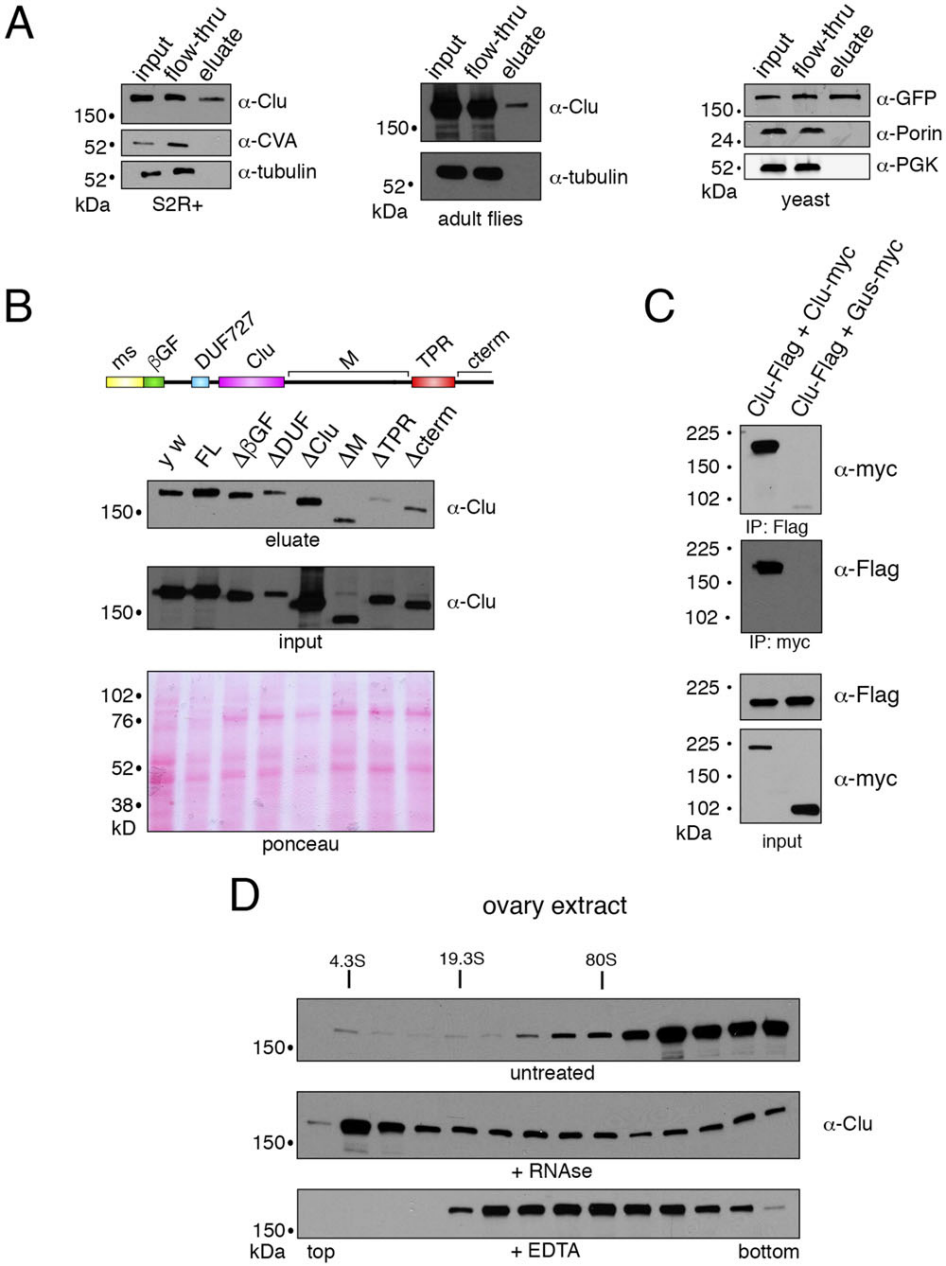
**The TPR domain of Clu facilitates mRNA binding.** (A) Westerns blots from S2R+ cells, adult flies, and yeast showing Clu and Clu1p-GFP bind mRNA. UV-irradiated extract (input) from each was exposed to oligo(dT) beads, washed (flow-thru), then the mRNA was eluted (eluate). Each fraction was probed with anti-Clu antibodies. Clu is present in both the flow-through and the eluate. Tubulin, CVA (ATP synthase) and phosphoglycerate kinase (PGK) serve as controls for Clu and Clu1p, respectively. (B) Clu lacking the TPR domain fails to associate with mRNA. Fly extract from *clu* mutant flies ectopically expressing deletion constructs under the control of UAS (*clu^d08713^*; *daGAL4*/UAS transgene) was exposed to oligo(dT) beads in the same way as in panel A. Only Clu lacking the TPR domain fails to interact with mRNA captured by the oligo(dT) beads (top blot). The western blot of the input indicates each construct is expressed at the correct predicted size. The Ponceau blot is included as a loading control. (C) Clu self-interacts. Western blots from transfected S2R+ cells showing Clu-Flag and Clu-myc reciprocally immunoprecipitate. Myc-tagged glucuronidase (GUS) was used as a control. (D) Western blots of fractions taken from 5-50% sucrose gradients. Extract from well-fattened ovaries shows Clu sediments in the heaviest fractions (top blot). Upon RNase treatment (middle blot), Clu shifts to the lighter fractions. Adding EDTA to dissociate ribosomes causes Clu to shift to lighter fractions (bottom blot). Approximate Svedberg units were based on molecular weight standards and RpL7a (Fig. S1).

For a more physiological assay, we expressed UAS-*clu* deletion constructs ectopically in a *clu* null mutant background using *daughterless*-*GAL4* (*daGAL4*) and assayed for rescue of the *clu* mutant phenotypes (Sen et al., 2013). Each construct appeared stably expressed at the correct size, though at different levels even though the transgenes are all inserted at the same locus in each fly stock (Fig. 3B, input). Previously we showed expressing *Drosophila FL*-*clu* and *cluh* rescues *clu* mutant deficits in fertility, negative geotaxis and germ cell mitochondrial mislocalization (Sen et al., 2015). Expressing *Drosophila FL*-*clu* and *cluh* also rescues *clu* mutant delayed pupation and adult survival (Fig. 2A-C). Larvae expressing ΔβGF, ΔDUF, ΔClu, ΔM, ΔTPR, and Δcterm domains pupate at the same rate as *clu* null mutant larvae, indicating each of these domains is important for pupation (Fig. 2A, dashed lines). Expressing UAS-*cluh* decreased the pupation delay (Fig. 2A, orange dashed vs white dashed), but did not return the rate to sibling wild type control levels (Fig. 2A, day 6 orange dashed line vs orange solid line, *P*≤0.0001). Cluh shares 53% amino acid identity with *Drosophila* Clu, but lacks the ms domain. *clu^d08713^*/CyO ActGFP larvae also exhibited a slight delay in pupation compared to the other wild type sibling controls, however, it does not rise to the level of statistical significance (Fig. 2A, white solid line vs all solid lines). In agreement with the pupation data, attempting to rescue the *clu* mutant adult lethality with ΔβGF, ΔDUF, ΔClu, ΔM, ΔTPR, and ΔC-term domains failed (Fig. 2B). The adults that hatched died within approximately four days and were as weak and sick as *clu* null adult flies (Fig. 2B; data not shown). Expressing Cluh in the *clu* null background substantially rescued the adult lethality, however there was adult death during the first week, after which adult mortality stabilized and the remaining flies exhibited a normal lifespan and slope of mortality (Fig. 2C).

To confirm that the lack of adult survival also affects mitochondrial output, we measured the amount of ATP in adults (Fig. 2D). *clu* null mutants had approximately twenty percent the amount of ATP when normalized to the ATP levels in *clu* heterozygous adults. Expressing *FL*-*clu* significantly increased the amount of ATP, but to less than half the level of the heterozygote (Fig. 2D). Although this represents a significant decrease in the amount of ATP, the lifespan, egg laying capacity and climbing ability are normal, indicating adults could tolerate substantially reduced levels of ATP (Fig. 2C,D) (Sen et al., 2013, 2015). Expression of *cluh* also significantly increased the amount of ATP over mutant levels, but to a lesser degree. Since we observed an initial, consistent die-off of adults within the first week post-eclosion, this suggests that the amount of ATP made in Cluh-expressing adults is partially insufficient during the first week of life, but is adequate if the adults can survive past that first week. Consistent with the lack of developmental rescue, expressing ΔBGF, ΔDUF, ΔClu, ΔM or ΔTPR domains in *clu* null adults did not increase ATP levels, and may have had a dominant negative effect since ATP levels are significantly reduced compared to *clu* null adults alone. *Superoxide dismutase 2* (*SOD2*) mutants, which we previously showed have decreased ATP levels, serve as a control (Sen et al., 2013). Taken together, our data from S2R+ cells and *in vivo* indicate that with the exception of the ms domain, all six identified domains when deleted singly are required for normal development and mitochondrial function.

### TPR domain of Clu facilitates mRNA binding

Clu1p and Cluh have previously been identified as ribonucleoproteins that directly bind the mRNA of nuclear-encoded mitochondrial proteins (Gao et al., 2014; Mitchell et al., 2013). However, it is not known whether *Drosophila* Clu also has this ability. In addition, the domain of Clu responsible for the mRNA interaction has not been identified. In order to do this, we took advantage of our deletion constructs. To show and verify, respectively, that *Drosophila* Clu and yeast C-terminally tagged Clu1p-GFP bind mRNA, we took extract from S2R+ cells, adult flies, and yeast and exposed the extract or cells to UV-irradiation, which creates covalent bonds between nucleic acid and proteins in close proximity (Fig. 3A, input). This UV-irradiated extract was then mixed with oligo(dT) beads to capture mRNAs. From this mixture, we found Clu and Clu1p-GFP in the flow-through of the wash, indicating much of it is not bound to mRNA. After eluting the mRNA from the beads, we found Clu and Clu1p present, as anticipated. ATP synthase (CVA), tubulin, and phosphoglycerate kinase (PGK) were used as controls and were only present in the input and flow-through.

Clu has several domains, yet does not contain a canonical RRM RNA-binding domain. Searching the sequence for potential RNA binding domains yielded two; however, the sequences are not well conserved between species. To test whether deleting any domain(s) of Clu caused a decrease in mRNA binding, we expressed each UAS-driven deletion construct with *daGAL4* in a *clu* null background, UV-irradiated the adult fly extract, and exposed the extract to oligo(dT) beads (Fig. 3B). Comparing the amount of each mRNA-bound Clu construct to the respective input, we found deleting the TPR domain caused a large decrease in the amount of bound mRNA. Deleting any of the other domains did not have as large an effect.

It was crucial to test the deletion constructs in a *clu* null background because we found Clu is able to form a complex with itself. By transfecting S2R+ cells with both myc- and FLAG-tagged full-length Clu constructs, we found Clu can reciprocally co-immunoprecipitate with itself (Fig. 3C). This interaction could be direct or indirect, and we do not yet know if Clu forms a dimer, tetramer or multimer, or whether the large Clu particles that can be seen in cells are a result of this interaction (Cox and Spradling, 2009; Sen et al., 2013, 2015).

### Clu sediments in the heaviest fractions *in vivo*

As assayed by immunofluorescence, fly tissues contain homogeneous cytoplasmic Clu, but also have prominent mitochodrially associated Clu particles, particularly in the female germ cells (Cox and Spradling, 2009; Sen et al., 2015). These particles are reminiscent of many different ribonucleoprotein particles found in the germline (Schisa, 2012). Using immunofluorescence after detergent extraction, Cluh is also found associated with mitochondria (Gao et al., 2014). Since Clu associates with proteins found in the mitochondrial outer membrane, and both Clu and Cluh are found in the mitochondrial fraction, we tested the sedimentation profile of Clu using sucrose gradients. Using ovary extract from well-fattened females to which cyclohexamide was added, we found Clu predominantly in the heaviest fractions (Fig. 3D). This is in contrast with Cluh, which is found in the light fractions in HeLa cell extract (Gao et al., 2014). Since Clu can bind mRNA, we exposed the extract to RNAse, and found a large portion of Clu shifted to the lighter fractions. These data support that Clu bound to RNA is found in the heavier fractions compared to Clu in the absence of RNA.

### Clu associates with ribosomal proteins

**Fig. 4.**
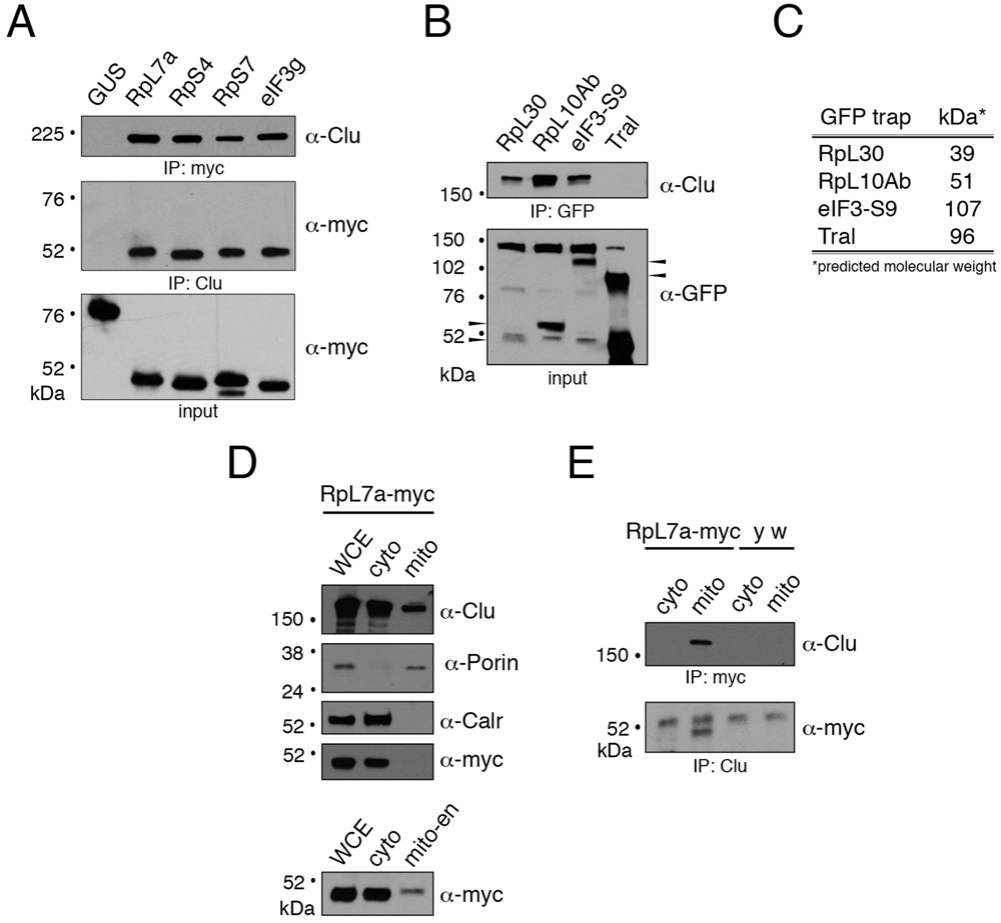
**Clu binds the ribosome at the mitochondrial outer membrane.** (A) Western blot of transfected S2R+ cells showing Clu reciprocally co-immunoprecipitates with myc-tagged large (RpL7a) and small (RpS4, RpS7) subunit ribosomal proteins as well as eIF3g. Myc-tagged plant glucuronidase (GUS) was transfected and used as a negative control. (B) Western blot of fly extract from adults expressing GFP-traps for RpL30, RpL10Ab and eIF3-S9 showing Clu can also form a complex with these three proteins. Pull-down from the GFP-trap for the ribonucleoprotein Trailerhitch (Tral) was used as a negative control. Arrowheads designate the correct size proteins. The other bands are non-specific. (C) Approximate molecular weights of each GFP trap. (D) Western blot showing whole cell extract (WCE), cytoplasmic (cyto) and mitochondrial (mito) extract fractions from adult flies expressing UAS-*RpL7a*-myc. RpL7a is present in the 4× enriched mitochondrial fraction (mito-en). Calr, Calreticulin. (E) After performing reciprocal co-immunoprecipitations on both cytoplasmic and mitochondrial fractions of the same extract, Clu is present in the mitochondrial fraction, but not in the cytoplasmic fraction. *y w* extract is used as a negative control. The upper bands in the anti-myc blot are nonspecific.

Clu1p interacts with eIF3 and eIF5 (Jao and Chen, 2006; Vornlocher et al., 1999). In addition, using bioinformatic analysis, Cluh was found to be co-regulated with genes involved with ribosomal biogenesis, RNA processing and translation (Gao et al., 2014). While these researchers also found Cluh co-sedimented with the ribosomal fraction from mouse liver, it is still unknown if Clu can form a complex with the ribosome (Gao et al., 2014). In order to identify Clu interacting proteins, we performed Clu immunoprecipitation (IP) followed by mass spectrometry on selected Coomassie stained bands that differed between Clu IP and control and found several ribosomal proteins (Table S2). To verify this preliminary data, we performed co-IPs with three of the identified ribosomal proteins. We transfected S2R+ cells with myc-tagged large Ribosomal protein (RpL) 7a, small Ribosomal protein (RpS) 4 and RpS7, performed reciprocal co-IPs and found Clu can form a complex with each (Fig. 4A). Myc-tagged GUS was used as a negative control. Yeast eIF3g/TIF35 was shown to bind Clu1p (Vornlocher et al., 1999). *Drosophila* has two paralogues of eIF3g, a and b (Lasko, 2000). We recapitulated the yeast result using myc-tagged *Drosophila* eIF3gb (also called eIF3-S9) (Fig. 4A).

The Rp–Clu interaction in S2R+ cells relies on over-expressed ribosomal proteins. To extend this analysis *in vivo*, we used extract from adult flies containing so-called GFP-traps for RpL30, RpL10Ab and eIF3-S9 (Buszczak et al., 2007). These fly stocks have GFP inserted into the intron of genes, which subsequently becomes spliced in frame into the resulting mRNAs. Thus, GFP expression is under control of the native promotor. Using anti-GFP antibody to pull-down the proteins, we found Clu present, even in traps with relatively low levels of GFP tagged protein (RpL30, for example) (Fig. 4B,C). The GFP trap for the ribonucleoprotein Trailerhitch (Tral) was used as a negative control (Wilhelm et al., 2005). Because both large and small ribosomal proteins can associate with Clu, we conclude that Clu associates with the ribosome. Since Clu sediments in an RNA-dependent manner in sucrose gradients, we tested if dissociating the ribosome using EDTA also caused a shift. After exposing ovary extract from well-fattened females to EDTA, we found there was indeed a shift towards the lighter fraction in the sedimentation pattern of Clu (Fig. 3D, top vs bottom panels).

### Clu physically associates with the ribosome at the mitochondrial outer membrane

We have previously established that Clu is found in the cytoplasm, and also binds to mitochondrial outer membrane proteins, including TOM20, porin, and PINK1 (Sen et al., 2015). To address if Clu binds ribosomal proteins associated with free ribosomes in the cytoplasm and/or those associated with mitochondria, we fractionated lysate from UAS*-RpL7a-myc* expressing adult flies and performed immunoprecipitations. At the same relative dilution, RpL7a is present in whole cell extract as well as the cytoplasmic fraction, but is very low and cannot be detected in the mitochondrial fraction (Fig. 4D, upper panels). However, RpL7a can be seen in four times enriched mitochondrial fractions (Fig. 4D, mito-en, lower panel). After performing reciprocal co-immunoprecipitations on both cytoplasmic and mitochondrial fractions, we found Clu preferentially associated with RpL7a in the mitochondrial fraction (Fig. 4E). Extract from wild type *y w* flies was used as a negative control. Mitochondria and the endoplasmic reticulum (ER) are very closely associated in the cell, even to the degree that they make direct contacts. To test if the Clu-RpL7a association in the mitochondrial fraction is due to residual contamination of ER-associated ribosomes, we probed the mitochondrial fraction with the ER membrane protein Calreticulin. While we cannot completely rule out the absence of ER, using our method of fractionation we found no Calreticulin in the mitochondrial fraction as judged by western blot (Fig. 4D).

### Ribosomal proteins are highly enriched in larval neuroblasts

**Fig. 5.**
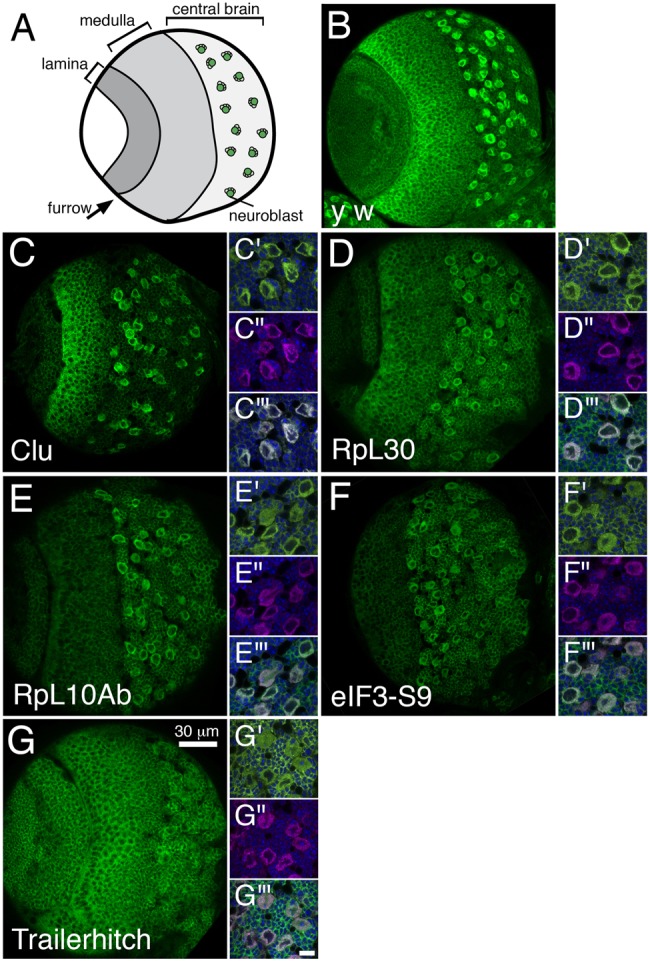
**Clu and proteins involved in translation are highly expressed in larval neuroblasts.** (A) Schematic of one lobe of a third instar larval brain. (B) Wild-type (*y w*) larval brain lobe labeled with anti-Clu antibody (green). (C-C‴) A GFP-trap for Clu (green, C,C′) mimics the staining pattern of Clu antibody (magenta, C″). C‴ is the merge. (D-E‴) GFP-traps for the large ribosomal subunit proteins RpL30 (green, D,D′) and RpL10Ab (green, E,E′) are highly expressed in neuroblasts and co-localize with Clu antibody (magenta, D″,E″). D‴ and E‴ are the merges. (F-F‴) Eukaryotic initiation factor 3-S9 (eIF3-S9) also has increased expression in neuroblasts (green, F,F′) which co-localizes with Clu antibody (magenta, F″ and merge F‴). (G-G‴) By contrast, the ribonucleoprotein Trailerhitch (Tral) is ubiquitously highly expressed in the larval brain (green, G,G′) and not concentrated in neuroblasts or with Clu antibody (magenta, G″ and merge G‴). Note the characteristic Tral ribonucleoprotein particles (green, G′). Green=anti-Clu (B), anti-GFP (C,C′,D,D′,E,E′,F,F′,G,G′), magenta=anti-Clu (C″,D″,E″,F″,G″). Scale bar=30 µm for main panels, 10 µm for all prime panels.

Previously, we found Clu is highly enriched in the larval stem cells called neuroblasts, as well as in cells in the medulla adjacent to the furrow separating the medulla from the lamina (Fig. 5A,B) (Sen et al., 2013). While proteins involved in translation might be expected to be ubiquitous, we found GFP-trap lines for ribosomal proteins for the large subunit were highly expressed in neuroblasts. RpL30 had a pattern highly reminiscent of Clu, with expression also in the medulla (Fig. 5D). RpL10Ab was enriched in neuroblasts, but had much lower expression in other regions of the brain lobe (Fig. 5E). In addition, eIF3-S9 was also enriched in neuroblasts, with lower expression in other brain lobe regions, similar to RpL10Ab (Fig. 5F). It is possible the high expression in neuroblasts of these GFP-trap lines is due to an artifact of using the GFP antibody. As a control, we used anti-GFP antibodies against dissected brains from Trailerhitch (Tral) GFP-trap larvae. These larval brain lobes had highly expressed protein in all cells types in the lobe (Fig. 5G). Discrete Tral puncta, like those seen in the ovary, could be observed at higher magnification, indicating the RNP aspect of Tral staining remains (Fig. 5G′) (Wilhelm et al., 2005).

## DISCUSSION

Clu is a large protein (∼160 kDa) that contains several domains. With the exception of the N-terminal ms domain, each domain when deleted fails to rescue the mitochondrial clumping phenotype in S2R+ cells, as well as *clu* mutant phenotypes *in vivo*. Given that Clu is able to associate with proteins involved in multiple different processes (ribosomal proteins, mitochondrial outer membrane proteins, and the PINK1–Parkin complex), Clu may act as a type of scaffold that can bring together several mechanisms in order to maintain mitochondrial function and output. Previously we showed lack of Clu causes an increase in mitochondrial oxidative damage and a decrease in ATP (Sen et al., 2013). Over-expressing single-domain deletions significantly reduced ATP levels below *clu* mutants alone (Fig. 2D). This could be because too much Clu can have a dominant negative effect by titrating out critical binding partners, thus negatively impacting mitochondrial function. Alternatively, high Clu levels may be toxic in and of themselves, however, overexpressing *FL*-*clu* does not appear to be toxic to flies.

While RNAi knockdown of Clu in S2R+ cells causes mitochondrial clumping, the cells themselves do not have reduced levels of ATP compared to control (Fig. 1G). This may be due to the differences in metabolism between cultured cells and cells *in vivo* (Moreno-Sanchez et al., 2007; Wallace, 2012). For example, there was a marked difference between the sedimentation profiles of Clu and Cluh. Cluh is found in the lighter fractions in extract from HeLa cells, whereas Clu from ovary extract was always found in the heaviest fractions in a sucrose gradient. HeLa cells, as well as many other transformed cultured cells, are known to use high amounts of glucose that appears to be used for glycolysis. For tumors, this is known as the Warburg effect (Warburg, 1956). In addition, glucose has been documented to inhibit oxidative phosphorylation, which is known as the Crabtree effect (Ibsen, 1961). In fact, the culture media often used, DMEM, contains pre-diabetic levels of glucose, thus providing the cells with high concentrations of glucose. The different metabolism and use of mitochondria could be one explanation for the discrepancy between the sedimentation of Clu and Cluh.

Previously, Gao et al. showed that Cluh co-sediments with light membranes and the ribosomal fraction and is released with high salt, suggesting it is a ribosome associated protein (Gao et al., 2014). We have extended this analysis to show that Clu reciprocally co-IPs with three RpLs and two RpSs, as well as two eIF3 proteins, and thus does indeed bind the ribosome. Furthermore, addition of EDTA, which dissociates ribosomes, caused Clu to shift from the heaviest to lighter fractions in a sucrose gradient. However, most importantly, we can show that Clu is able to bind RpL7a in the mitochondrial fraction but not in the cytoplasm. While there are high levels of homogeneous Clu in the cytoplasm, Clu particles in *Drosophila* germ cells are clearly juxtaposed to mitochondria but do not co-localize (Cox and Spradling, 2009; Sen et al., 2015). As we previously showed, Clu associates with mitochondrial outer membrane proteins (Sen et al., 2015). These data taken together support that Clu predominantly binds the ribosome at the outer mitochondrial membrane *in vivo*. In addition, Clu associates with TOM20, thus placing Clu in the same location at which mitochondrial protein import occurs (Sen et al., 2015).

*Drosophila* third instar neuroblasts divide frequently (approximately every twenty five minutes) to ultimately create the adult brain. Components of the Myc pathway, which is responsible for ribosome biogenesis, have been shown to have differential expression in neuroblasts (Betschinger et al., 2006; Kim et al., 2000; Lee et al., 2006). The nucleolar protein Mushroom body miniature (Mbm), which is highly expressed in neuroblasts, is a transcriptional target of Myc and is involved in ribosome biogenesis (Hovhanyan et al., 2014). Conversely, Brain tumor (Brat) has been shown to be a negative regulator of Myc and becomes asymmetrically localized away from the neuroblast into the daughter cell during cell division (Betschinger et al., 2006; Frank et al., 2002; Lee et al., 2006). Work involving transcriptome analysis found that genes involved in ribosome biogenesis, as well as other processes, are highly represented in neuroblasts compared to differentiated neurons (Berger et al., 2012). Given that an increase in ribosome biogenesis appears intrinsically important for neuroblasts, it follows that ribosomal proteins should also be enriched in neuroblasts. Our work here shows for the first time, to our knowledge, that RpLs, as well as eIF3-S9, are highly enriched in neuroblasts (Fig. 5).

Clu1p and Cluh are ribonucleoproteins, however which Clu domain binds mRNA has not been identified (Gao et al., 2014; Mitchell et al., 2013). Here, we show the TPR domain of Clu is critical for mRNA binding. TPR domains are well known to facilitate protein-protein interactions (D'Andrea and Regan, 2003). Proteins containing the related HAT domain (half a TPR) and pentatricopeptide repeats (PPRs) have been shown to directly bind RNA (Hammani et al., 2012; Williams-Carrier et al., 2008). HAT and PPR domains have similar sequence and structure to TPRs. Since UV-cross linking causes covalent bonds between nucleic acid and protein, our data showing deletion of the TPR greatly decreases the amount of bound mRNA is consistent with it potentially directly binding mRNA (Small and Peeters, 2000; Williams-Carrier et al., 2008).

Ours, and previously published data, strongly support that Clu normally binds to mRNA *in vivo*. Combined with our data showing Clu binds ribosomal proteins, and can do so in the mitochondrial fraction, suggests that the role of Clu in the cell is to facilitate mRNA binding and association with the ribosome at the mitochondrial outer membrane. In fact, mitochondrial protein levels are globally reduced in *Drosophila clu* mutants, and specific mitochondrial proteins encoded by Cluh-bound mRNAs are reduced in *cluh* knockout immortalized mouse embryonic fibroblasts (Gao et al., 2014; Sen et al., 2015). Thus, Clu may act to provide site-specific translation or co-translational import of mitochondrial proteins. The molecular mechanism of co-translational import is well-established for endoplasmic reticulum bound proteins (Nyathi et al., 2013). It is increasingly clear that co-translational import occurs on mitochondria, however the mechanism is not as clear (for recent review see Lesnik et al., 2015). Clu forms a complex with the mitophagy proteins PINK1 and Parkin, and PINK1 and TOM20 have been implicated in localized translation of mRNAs encoding respiratory chain proteins (Gehrke et al., 2015). It is possible that Clu may function to link lack of mitochondrial import and activity with mitochondrial damage sensing and destruction (Sen et al., 2015).

## MATERIALS AND METHODS

### Fly stocks

*clueless^d08713^*/CyO Act GFP and *y w*; *clueless^CA06604^* were described previously (Cox and Spradling, 2009). The GFP traps *y w*; *RpL30^CB03373^*, *y w*; *RpL10Ab^CB02653^*, *y w*; *eIF3-S9^CB04769^*, and *y w*; *trailerhitch^CA06517^* were provided by Allan Spradling (Buszczak et al., 2007). *w^1118^*; *UASp FL-clu* and *w^1118^*; *UASp cluh* are described in Sen et al. (2015). y^1^ w*; Sod2^Δ02^/CyO and *daughterless-Gal4* were obtained from the Drosophila Stock Center, Bloomington, MD, USA. For wild type, *y^1^ w^67g23^* was used. Flies were reared on standard cornmeal fly media at 22°C or 25°C.

### Constructs

Full length open reading frames were amplified from the following *Drosophila* ESTs (Drosophila Genomics Resource Center, Bloomington, MD, USA): *clu* (RH51925), *RpL7a* (RE05022), *RpS4* (RE57333), *RpS7* (RE18653), and *eIF3g* (LD24347). Full length *clu* and Δ*ms* were sub-cloned into a pENTR/D-TOPO vector from cDNA clone (RH51925) using a pENTR/D-TOPO cloning kit (Invitrogen). All other *clu* deletion constructs were made using a QuickChange lightning Site-Directed Mutagenesis Kit (Agilent Technologies) using the Clu full length clone as template. Primers used for the cloning purpose are presented in Table S1. Entry clones were swapped into respective Gateway vectors (The Drosophila Gateway Collection, Carnegie Institution of Washington, USA) using Gateway LR Clonase II kit (Invitrogen).

### Transgenic flies

To create *w^1118^*; *UASp-cluΔClu*, *w^1118^*; *UASp-cluΔTPR*, *w^1118^*; *UASp-cluΔβGF*, *w^1118^*; *UASp-cluΔDUF*, *w^1118^*, *UASp-cluΔM*, *w^1118^*; *UASp-cluΔcterm* stocks, *clu* constructs were cloned into a modified pPW destination vector (The Drosophila Gateway Collection) containing phiC31 attB sites (gift from Dr Michael Buszczak, University of Texas Southwestern) and commercially injected and transformed using phiC31 integrase into the 68A4 landing site on the third chromosome (Genetic Services, Cambridge, MA, USA). For S2R+ tissue culture constructs, *clu* constructs were cloned into pAWM containing a C-terminal myc tag (The *Drosophila* Gateway Collection).

### Pupation and adult survival measurements

Pupation and adult survival assays were performed as described (Sen et al., 2013). *P*-values were calculated in Excel (Microsoft) using a two-tailed Student's *t*-test.

### Immunofluorescence

Wandering third instar larval brains were dissected and fixed as previously described (Sen et al., 2013) with the following modification. Larval brains were labeled sequentially using: guinea pig anti-Clu N-terminus (1:2000; Cox and Spradling, 2009), donkey anti-guinea pig Cy3 (1:500, Jackson ImmunoResearch, West Grove, PA, USA), rabbit anti-GFP (1:2000, Torrey Pines Biolabs, Secaucus, NJ, USA), and donkey anti-rabbit Alexa 488 (1:500, Jackson ImmunoResearch, West Grove, PA, USA), with 3 times, 10 min washes (1× PBS:0.1% Triton X-100:1% BSA) in between each antibody. S2R+ culture cells were fixed and mounted as described (Sen et al., 2013). The following primary antibodies were used: mouse anti-Complex V (1:1000, cat# MS507, Mitosciences, Eugene, OR, USA) and rabbit anti-GFP (Torrey Pines Biolabs). The following secondary antibodies were used: goat anti-mouse IgG_2b_ Alexa 488, and goat anti rabbit Alexa 568 (Molecular Probes, Life Technologies, Grand Island, NY, USA). Images were collected using Zeiss 710 and Zeiss 700 confocal microscopes and 63× Plan Apo NA 1.4 lens.

### Western blotting and immunoprecipitations

Western blotting, immunoprecipitations (IP), and cell fractionations were performed as described (Sen et al., 2013, 2015). IPs from mitochondrial and cytoplasmic fractions were performed as described, except the IP was done in mitochondrial isolation buffer containing 0.5% Triton X-100 and 1 mM DTT. Western blots were exposed to the following antibodies: anti-Clu (1:15,000; Cox and Spradling, 2009), anti-alpha-Tubulin (1:5000, cat# AA4.3-S, Developmental Studies Hybridoma Bank, University of Iowa, IA, USA), anti-Myc (1:5000, cat# M4439, Sigma-Aldrich), anti-Complex V (CVA) (1:100,000, cat# Ab14748, Mitosciences), rabbit anti-GFP (1:10,000, cat# Ab290, AbCam), anti-PGK (1:25,000, cat# Ab113687, Invitrogen), mouse anti-FLAG (1:10,000, cat# F1804, Sigma), rabbit anti-Calreticulin (1:1000, cat# Ab2907, Abcam), mouse anti-Porin (1:2000, cat# Ab14734, Mitosciences).

### Mass spectrometry analysis

Immunoprecipitation was performed from S2R+ cell extract using anti-Clu antibody, with guinea pig IgG as a control. Both samples were run side-by-side using standard SDS-PAGE on a 4-15% polyacrylamide gel. After staining the gel using Colloidal Blue, we identified three clearly unique bands that were present in the anti-Clu antibody lane and absent in the control (data not shown). One high molecular prominent band which migrated at the same size as Clu was cut and analyzed as a positive control. Two other bands were cut from the gel (∼38 kDa and ∼30 kDa) and sent for mass spectrometric analysis using NanoLC-ESI-MS/MS peptide sequencing technology (ProtTech, PA, USA).

### Sucrose gradients

Dissected ovaries and S2R+ cells were homogenized in mDXB (25 mM HEPES pH 6.8, 50 mM KCl, 1 mM MgCl_2_, 125 mM Sucrose, 1 mM DTT and 1× protease inhibitor cocktail) using a 1 ml Dounce homogenizer. Extract was clarified by spinning at 2000 ***g*** for 10 min. Supernatant was divided into two tubes. RNase inhibitor (Ambion) was added to one tube and RNAse A was added to the other tube and incubated on ice for 15 min. Samples were loaded onto sucrose step gradients (5-50% in 5% increments, made in mDXB) and centrifuged at 235,000 ***g*** for 2 h in a Beckmann ultracentrifuge. Fifteen fractions of 330 µl each were collected starting with the top lightest fraction. Proteins were concentrated using methanol-chloroform extraction method.

### ATP assay

ATP assays were performed on adult flies as described previously (Sen et al., 2013). Each sample was processed in duplicate and read in duplicate. The amount of ATP was normalized against protein concentration.

### S2R+ cell RNAi

Clu RNAi knockdown and mitochondrial mislocalization analysis were performed as described previously (Sen et al., 2015). 160 or greater cells were examined for each construct. For Fig. 1E-G, each experiment was performed in technical triplicate. The S2R+ cell were purchased from Drosophila Genomics Resource Center, stock # 150.

### RNA binding

Chilled cell suspension and yeast (Clu1p-GFP, Thermo Fisher Scientific) were UV irradiated 2× at 250 mJ power setting in a Stratalinker 1800 (Stratagene). Material from 50 adult flies crushed in 400 μl H_2_O using a blue pestle and Eppendorf tube (Kimbel Chase Life Science) was placed in the bottom of a well of a 12-well dish (BD Falcon) and irradiated as described above. This type of relatively gentle manipulation should not result in extensive cell lysis. Following irradiation, each sample was lysed in RNA lysis buffer (RLB) [20 mM HEPES pH 7.4; 50 mM KCl, 1% Triton X-100, 0.5% NP-40 (sub), 10 mM EDTA, 0.5 mM EGTA, 5% glycerol, 0.5% sodium deoxycholate, 0.1% SDS, 2 mM DTT, 1× Protease inhibitor cocktail, 40 U/ml RNase inhibitor (Ambion)]. Samples were cleared by spinning at 16,000 ***g*** and incubated with Oligo d(T)25 magnetic beads (NEB) for 2 h. After incubation, the beads were separated on a magnetic rack and washed 2×10 min with Wash Buffer [RLB containing 0.2% SDS with the RNase inhibitor substituted with 1× ProtectRNA Rnase inhibitor (Sigma)]. RNA was eluted by incubating the beads with warm TE for 5 min, then the beads were separated from the supernatant with a magnetic rack. The eluted samples were used for western blotting. The lysate from S2R+ cells and yeast was the same except without SDS and deoxycholate in the Wash Buffer.
